# Medical News

**Published:** 1853-12

**Authors:** 


### MEDICAL NEWS.

### DEATH FROM CHLOROFORM DURING ITS ADMINISTRATION PREVIOUS TO AN OPERATION FOR HERNIA.

[Under the care of Mr. QUAIN.]

Mr. Hillier, the resident Medical officer, has favored us with the following particulars of a case of death from chloroform which occurred recently. The administration of the drug was, as usual, under the care of Mr. Hillier, according to his appointment at the hospital. It will be seen, that the patient had been of intemperate habits; and, as the autopsy revealed, the subject of extensive fatty degeneration of the muscular structure of the heart, and that, previous to the administration, she had been nearly three day's ill, fasting, exhausted, and suffering ultimately from acute peritonitis. These considerations make the fatal result quite comprehensible.

*Particulars, etc.*—E. R., aged 40, a woman of moderate height, rather thin. Her general health has been pretty good; she had not been liable to palpitation or dyspnœa. Had been in the habit of drinking pretty freely. Admitted on October 5, at 11 p. m. She was suffering from the symptoms of strangulated femoral hernia, which had existed two days and a-half. Efforts were made to reduce the hernia, both without and with a warm bath, in which she was for upwards of half an hour, without getting very faint. These efforts being unsuccessful, an operation was at once determined on. Her pulse was at this time regular, and of tolerable strength.

Chloroform was administered in the usual way, on a piece of lint, which was held at first three or four inches from the patient's face, and and then brought to within an inch and a-half of her nose and mouth, leaving space around for the admission of air.

For three or four minutes nothing unusual presented itself; the pulse and respirations proceeded normally. There was put on the lint, at first, one fluid drachm of chloroform; and, at the end of three or four minutes, 40 minims more were added. This was the whole quantity of chloroform employed. Within a minute after the second quantity of chloroform was added; the patient struggled violently both with her arms and legs. During these struggles I was holding her right hand, and was unable to feel the pulse, in consequence of her constant motions. The struggling lasted about a minute, and, on its ceasing, the patient commenced to breathe with loud, rough stertor. I at once removed the lint from before the face, and felt for the pulse, which I could not find. Immediately cold water was dashed on to her face. She breathed with this stertor for two or three short inspirations, and then two or three long ones, and then breathing ceased. Immediately artificial respiration was resorted to, and within a minute galvanism was applied to the back of her neck and the diaphragm. Under the influence of these agencies, the patient gasped about three times at intervals ; after this no further signs of life were exhibited.

Tracheotomy was resorted to at the end of a few minutes, and the artificial respiration continued through this opening for about three-quarters of an hour.

At the time when the stertor commenced and the pulse failed, the pupils were dilated, and the face of the patient was only slightly altered. Her tongue was not retracted, for one of the bystanders at once put his fingers into her mouth to ascertain this.

Chloroform from the same bottle had been administered by me on the same day to five patients, in the same manner, without any unpleasant results.

*Autopsy made by Dr. Garrod, thirteen hours after Death.*—Rigor mortis well-marked in all the limbs. The blood very fluid in all parts of the body.

Abdomen very tympanitic ; diaphragm extends up to opposite the fourth rib on the left side, and to third interspace on the right. About an ounce of colorless fluid in the pericardium. The heart quite collapsed and empty,—this may have been due in some measure to the fluidity of the blood.

Anterior aspect of heart covered with fat, muscular substance being visible at one or two points only.

Weight of heart, 7¾ oz. ; valves healthy. Walls of right ventricle flabby and pale ; mean thickness, one-eighth of an inch. At some parts, the muscular substance is in a very thin layer, being much encroached on by fat. In several places, there is scarcely any muscular fibre visible. This is chiefly the case near the apex. Examined by the microscope, much fatty degeneration of the muscular fibres of the right ventricle generally was discovered.

Wall of left ventricle flabby, dry in appearance and pale ; very friable.

Old adhesions on both pleuræ. No appearance of atheroma in thoracic aorta. Both lungs cripitant throughout ; not much engorged.—Brain not congested. The arachnoid exhibited marks of chronic thickening and opacity.

The intestines, above the strangulation, were much distended with flatus, and inflamed. The strangulated portion was of a very dark color, and had blood effused into its coats.

Liver, kidneys, and spleen normal.

[*Note.*—We are sorry to have to state, that another death from chloroform has occurred since the above was written. The patient, a woman, aged 19, suffering from rodent ulcer of the vagina, under the care of Mr. Paget, in St. Bartholomew's Hospital, died on Thursday afternoon. The intended operation was the application of the actual cautery. We shall give further particulars next week.]—*London Medical Times, October* 22, 1853.

DEATH UNDER CHLOROFORM.—About a fortnight ago, while the students were assembled in the operating theatre of the Infirmary, to see Mr. Syme's operation of perineal section performed by Dr. Dunsmure, and while chloroform was being administered preparatory to the operation, the patient suddenly expired. Every expedient that was possible under the painful circumstances was immediately resorted to by Dr. Dunsmure and his colleagues, but, unfortunately, without effect. I cannot but think, that a great error in the administration of chloroform prevails in the hospital here. Sufficient precautions are not used to allow a free admixture of atmospheric air with the anæsthetic. When this is attended to, the patient can be put under the influence of the drug more easily and with less suffering to himself, and can be kept under it for many hours consecutively. In hospital practice its administration is commonly entrusted to some raw dresser, who thinking he cannot give enough of a good thing, squeezes a towel saturated with it over the mouth, where it is kept firmly applied, so that the admixture of chloroform with atmospheric air is rendered impossible.—*Ibid, October* 15*th.*

